# Cardiac Sympathetic Activity and the Neuro-Humoral Theory on Heart Failure with Reduced Ejection Fraction: Have We Learned Enough?

**DOI:** 10.5935/abc.20180148

**Published:** 2018-08

**Authors:** Thiago Quinaglia A. C. Silva, Otávio R. Coelho-Filho

**Affiliations:** Faculdade de Ciências Médicas - Universidade Estadual de Campinas, São Paulo, SP – Brazil

**Keywords:** Heart Failure, Primary Dysautonomies, Chagas Cardiomyopathy, Myocardial/radionuclide imaging, ^123^I-MIBC

Since the 1970s,^1^^,^^2^ in observational trials, and more recently in placebo-controlled randomized trials,^3^^,^^4^ antagonizing beta-1 adrenergic receptors in the myocardium has been shown to mitigate the burden of heart failure with reduced ejection fraction (HFrEF). Mortality has been reduced by 34-65% according to these studies. However, inhibiting the action of the peripheral sympathetic nervous system (SNS) by blocking alpha1 or stimulating alpha-2 central receptors has shown negative results or even an increased mortality, despite reducing norepinephrine plasma levels.^5^^-^^7^ This indicates that SNS effects on myocardial receptors, more than in peripheral receptors, play a pivotal role in HFrEF pathophysiology.

Hyper-activation of the SNS and the renin-angiotensin-aldosterone axis, combined with an increase in load-dependent peptides and inflammatory cascades constitute the neuro-humoral theory on HFrEF progression. While the neuro-humoral response counteracts and compensates for an initial myocardial insult, in the long-term it contributes to the progression of the disease to the point that cardiovascular homeostasis eventually succumbs if not treated properly. Therapies targeting all these neuro-humoral responses have dramatically changed the natural history of HFrEF.

Even though neuro-humoral theory has scaffolded for treatment of heart failure, the same has not occurred for HFrEF diagnosis. Except for the (still) scant use of brain natriuretic peptide (BNP), diagnosis and follow-up of such patients has been largely based on the estimation of ejection fraction (EF). This parameter is obviously of great importance, but in addition to being highly variable (inter-observer variability can be as high as 13%),^8^ EF reduction occurs late in disease progression^9^ when intervention is often less efficacious. Thus, new methods that comprise early detection of myocardium at risk and allow response to treatment assessment are desirable.

Assessment of cardiac sympathetic activity can, in theory, fulfill these criteria. Iodine^123^– metaiodobenzylguanidine (^123^I-MIBG) scintigraphy is a well-known method to assess SNS cardiac activity and, though not widely applied, it can provide valuable information regarding early myocardial damage^10^ and response to beta1- adrenergic receptors blockade.^11^ In this issue of *Arquivos Brasileiros de Cardiologia*, Marino et al.^12^ present a study with an instigating design and shed light on how cardiac sympathetic dysfunction occurs in HFrEF patients. In the study, treated patients with Chagas’ cardiomyopathy appear to have similar sympathetic cardiac dysfunction compared to other treated HFrEF patients. These results could highlight the fact that treatment efficacy does not vary across HFrEF groups. Along with the current published scientific literature it is possible to speculate that cardiac Chagas disease begins with sympathetic denervation, evolves to perfusion disturbances and terminates in motility impairment.^13^^,^^14^ Intervention with optimized medical treatment could, then, delay the progression to terminal disease. Also, the manuscript shows that sympathetic function in HFrEF treated patients is still below normal thresholds described in previous studies, thus suggesting a possible residual risk related to SNS hyper-activation.

The study by Marino et al.^12^ is timely because it reminds clinicians and researchers that in order to maintain progress in treatment improvement and disease prevention it is of utmost necessity to keep track of its pathophysiology. Hyper-activation of SNS, renin-angiotensin-aldosterone axis and inflammatory cascades, among others, are cornerstones of the disease. Novel biomarkers to evaluate early myocardium at risk are highly needed, and some interesting ones seem to be in the pipeline. Late gadolinium enhancement, extracellular volume fraction and myocyte size quantification, assessed by cardiac magnetic resonance, as well as myocardial strain imaging by echocardiography and ^123^I-MIBG global and regional cardiac scintigraphy by nuclear imaging are promising methods, but these novel methods require validation in larger cohorts and in controlled clinical trials. Established and novel methods can be then integrated to provide a thorough evaluation of the HFrEF patient and perhaps reduce even more the burden of such ominous disease.
